# Additive Synergism between Asbestos and Smoking in Lung Cancer Risk: A Systematic Review and Meta-Analysis

**DOI:** 10.1371/journal.pone.0135798

**Published:** 2015-08-14

**Authors:** Yuwadee Ngamwong, Wimonchat Tangamornsuksan, Ornrat Lohitnavy, Nathorn Chaiyakunapruk, C. Norman Scholfield, Brad Reisfeld, Manupat Lohitnavy

**Affiliations:** 1 Center of Excellence for Environmental Health & Toxicology, Faculty of Pharmaceutical Sciences, Naresuan University, Phitsanulok, Thailand; 2 Pharmacokinetic Research Unit, Faculty of Pharmaceutical Sciences, Naresuan University, Phitsanulok, Thailand; 3 Department of Pharmacy Practice, Faculty of Pharmaceutical Sciences, Naresuan University, Phitsanulok, Thailand; 4 Center of Pharmaceutical Outcomes Research, Department of Pharmacy Practice, Faculty of Pharmaceutical Sciences, Naresuan University, Phitsanulok, Thailand; 5 School of Pharmacy, Monash University Malaysia, Selangor, Malaysia; 6 School of Pharmacy, University of Wisconsin-Madison, Madison, Wisconsin, United States of America; 7 School of Population Health, University of Queensland, Brisbane, Australia; 8 Department of Chemical and Biological Engineering, Colorado State University, Fort Collins, CO, United States of America; University of Cincinnati College of Medicine, UNITED STATES

## Abstract

Smoking and asbestos exposure are important risks for lung cancer. Several epidemiological studies have linked asbestos exposure and smoking to lung cancer. To reconcile and unify these results, we conducted a systematic review and meta-analysis to provide a quantitative estimate of the increased risk of lung cancer associated with asbestos exposure and cigarette smoking and to classify their interaction. Five electronic databases were searched from inception to May, 2015 for observational studies on lung cancer. All case-control (N = 10) and cohort (N = 7) studies were included in the analysis. We calculated pooled odds ratios (ORs), relative risks (RRs) and 95% confidence intervals (CIs) using a random-effects model for the association of asbestos exposure and smoking with lung cancer. Lung cancer patients who were not exposed to asbestos and non-smoking (A-S-) were compared with; (i) asbestos-exposed and non-smoking (A+S-), (ii) non-exposure to asbestos and smoking (A-S+), and (iii) asbestos-exposed and smoking (A+S+). Our meta-analysis showed a significant difference in risk of developing lung cancer among asbestos exposed and/or smoking workers compared to controls (A-S-), **odds ratios** for the disease (*95% CI*) were (i) **1.70** (A+S-, *1*.*31–2*.*21*), (ii) **5.65;** (A-S+, *3*.*38–9*.*42*), (iii) **8.70** (A+S+, *5*.*8–13*.*10*). The additive interaction index of synergy was 1.44 (95% CI = *1*.*26–1*.*77*) and the multiplicative index = 0.91 (95% CI = *0*.*63–1*.*30*). Corresponding values for cohort studies were 1.11 (95% CI = *1*.*00–1*.*28*) and 0.51 (95% CI = *0*.*31–0*.*85*). Our results point to an additive synergism for lung cancer with co-exposure of asbestos and cigarette smoking. Assessments of industrial health risks should take smoking and other airborne health risks when setting occupational asbestos exposure limits.

## Introduction

Lung cancer is responsible for 20% of all global cancer deaths. Its latency period is long (~20 yr) and survival rate poor (10%) [1]. Meta-analyses of epidemiological studies demonstrated that smoking had a strong relationship with lung cancer [2,3] and 70–90% of lung cancer patients are directly attributed to cigarette smoking [4]. Several compounds in tobacco smoke are classified as human carcinogens (Group 1) by the IARC including tobacco specific nitrosamines and benzo(a)pyrene, a carcinogenic polycyclic aromatic hydrocarbon [4,5]. Second-hand smoke also increases the risk of developing lung cancer by an estimated 25% in by-standers [6]. Besides smoking, other risk factors for lung cancer are arsenic, particulates from diesel engine exhausts, radon, and exposure to asbestos and other mineral fibers, [7,8].

Asbestos is a group of naturally occurring silicate mineral fibers widely used in building materials, vehicle brakes and thermal insulators since the 1900s. Asbestos types are classified according to their structures, chemical composition and thermal stability. Chrysotile or white asbestos (mainly Mg_3_(Si_2_O_5_)(OH)_4_) [9,10] accounts for most current use where asbestos is permitted while amosite (brown) and crocidolite (blue asbestos), belonging to the amphibole class, are stronger, more durable, and more heat resistant than chrysotile. There are many well documented lung disease cases in asbestos factory workers and miners from 1900 onwards [11–15]. The most common asbestos-associated diseases are benign pleural disease, asbestosis, lung carcinoma (small cell, squamous, and adenocarcinoma) and mesothelioma [16]. Mesothelioma has a very high association with asbestos exposure but otherwise uncommon [17]. It has high incidences among males of western countries and Japan where it is projected to peak between 2012 and 2030, a latency of 40–50 years after the peak use of asbestos during the 1930s-1970s [18].

Numerous studies have shown a clear association between carcinogenesis and either smoking or asbestos. However, associations may result from independent and unrelated mechanisms and therefore show additive effects while effects greater than summed individual actions implies biological interactions [19,20]. This is commonly referred to as synergism [21] but additive synergism is more appropriate. Conversely, a smaller effect than the sum of effects may be due to antagonistic interactions. Synergism might, less commonly, be multiplicative due to different types of interaction, for example where an effect requires the activation of two or more serial processes. Such distinctions are important for both possible treatment considerations and public health such as identifying those at greatest risk of disease. Some authors have sought to assess interactions between asbestos and smoking on lung cancer [22,23], and found the effects to be additive [24], more than additive [25] and multiplicative [26,27]. In animal experiments, co-exposure to asbestos and cigarette smoke also found contradictory interaction models [28–30]. Two previous meta-analyses [31,32] found associations between asbestos exposure and smoking for increased lung cancer risk and that the two carcinogenic effects were greater than the sum of their separate actions but again failed to agree on the type of interaction (multiplicative or additive). These reviews had some weakness (assessing individual interactive effects in each study and could not explain the dose-response for asbestos exposure). Also, they have been superseded by additional studies which relate asbestos exposure with smoking and lung cancer [22–27]. Besides increasing the power and weight of the data, these later studies were better designed and controlled, especially the Markowitz et al. study [24], and therefore better able to resolve these issues. Thus, we incorporated this data into a new systematic review and meta-analysis. We anticipate that such a study will better inform the risk assessment process in developing nations where most male semi-skilled workers are smokers, and occupational asbestos exposure continues to pose a health risk in populations where lung disease is a leading cause of mortality [33].

## Methods

The study was conducted and reported using the PRISMA (S1 PRISMA Checklist) [34] and MOOSE [35] guidelines.

### Search Strategy and study selection

We searched titles and abstracts PubMed, Embase, Scopus, ISI Web of Knowledge, and TOXLINE databases from their inception to May 2015. Combinations of the following key words were used: asbestos, crocidolite, amosite, chrysotile, tremolite, actinolite, anthophyllite, cigarette, cigarette smoke, cigarette smoking, pipe, cigar, tobacco, tobacco smoking, lung cancer, mesothelioma, lung carcinoma, and lung adenocarcinoma. There was no language restriction. Additional studies were also hand-searched from bibliographies of the selected studies.

### Inclusion and exclusion criteria

Studies were included if they met all of the following criteria: (1) original articles published in peer-reviewed journals; (2) human studies; (3) observational studies; (4) studies investigating associations between asbestos exposure and smoking with lung cancer, and; (5) studies reporting sufficient data for calculating odds ratios and relative risks. The studies not meeting the inclusion criteria described above were excluded. If there were duplicate populations, only the studies providing the most details, grater number of participants, followed populations for longer follow-up periods, or the most recently published were selected for meta-analysis. Two reviewers (YN, WT) independently appraised titles and abstracts retrieved from the comprehensive searches. The controversial reviews were discussed and resolved by a third reviewer (OL). If further details were required, the reviewers contacted the authors for more information.

### Data Abstraction and Quality Assessment

Information extracted from each study included first author, publication year, geographic area, study type (hospital-based case-control, population-based case-control, nested case-control, retrospective cohort, prospective cohort, and cross-sectional), total number of cases, and controls, fiber type (chrysotile, crocidolite, tremolite), industry type, measurement of asbestos and/or smoking exposure, asbestos exposure assessment method, definition of asbestos exposure and/or smoking, period of employment/exposure, measurement method (asbestos exposure, smoking), and classification of outcome. The Newcastle-Ottawa quality assessment scale (NOS) was used to assess the quality of the selected observational studies. The categories of NOS was based on selection of participants, comparability of study groups, and the exposure of interest (case-control studies) or outcome of interest (cohort studies) [36]. When each category is satisfied it attracts one or sometimes two ‘star(s)’ and a maximum of 9 stars for either case-control or cohort study, indicates the highest quality study [37].

### Statistical Analysis

Asbestos exposure was arbitrarily taken as more than 100 air-borne fiber-yr/ml of environmental air for >5% of their work time and cigarette smoking was categorized as smokers who smoked >15 cigarettes/day. Those subjects having lower and shorter fiber exposures and lower cigarette consumption were deemed as non-exposed or non-smokers, respectively.

Using the above cut-offs, subjects were placed into four groups: (1) those people not exposed to asbestos and non-smokers were classified as not exposed to asbestos and non-smoking (A-S-), (2) workers exposed asbestos and non-smokers were classified as asbestos-exposed and non-smoking (A+S-), (3) those not exposed to asbestos but smoked were grouped as non-exposed to asbestos and were smokers (A-S+), and (4) workers exposed to asbestos and smoked were classified as asbestos-exposed and smokers (A+S+). The primary outcome of the pooled analysis focused on comparing the summary effect of lung cancer risk in people without asbestos exposure and non-smoking versus co-exposure to asbestos and/or smoking as follows: (i) A+S- compared with A-S- (ii) A-S+ compared with A-S-, and (iii) A+S+ compared with A-S- and interaction between asbestos and smoking were evaluated using the Rothman Synergy Index [38]. Summary effect estimates were assessed discretely by averaging the natural logarithmic OR and/or RR weighted by their inverse variances. The pooled effect estimates were calculated using a random effects model by the method of DerSimonian and Laird [39]. Heterogeneity among selected studies was determined using the Q-statistic and *I-*squared tests [40]. *I-*squared (*I* ^*2*^) values of 25%, 50%, and 75% represented low, moderate, and high degrees of heterogeneity, respectively [41]. The meta-analysis of case-control and cohort studies were conducted separately due to differences in the nature of study design [42].

Subgroup analyses were performed according to the geographic area (Europe, America, others), asbestos type, study design (hospital or population, retrospective, prospective), and stratification of smoking level were used to assess the impacts of study characteristics on outcomes. Publication bias was quantified using funnel plot, Begg’s test and Egger’s test, where *p*>0.05 for both tests was considered to have no significant publication bias [43,44]. All analyses were performed using STATA software V.10.1 (Stata Corp, College Station, TX, USA).

### Determination of interactive effect

For measurement of interaction, there are 2 models to calculate this: the additive and the multiplicative scales. If these yield more than additive and multiplicative, there is a positive interaction. If less than additive/multiplicative, it is referred to as a negative interaction. The word “synergistic” means the effect two exposures is greater than the combined effect of each exposure. Thus, the value of interaction is more than either the additive or the multiplicative scales as appropriate, i.e., either additive or multiplicative synergism.

The joint effect of exposure to asbestos and smoking was first examined by estimating odds ratio (ORs) and relative risk (RRs). To determine whether co-exposure to asbestos and smoking is an additive and multiplicative scale, the synergy (*S*) and multiplicative (*V*) indices were calculated as follow [38,45].

Synergy index (*S*)

$$\begin{array}{l} S=\frac{X_{AS}-X_{0}}{X_{A}+X_{S}-2X_{0}} \end{array}$$

Multiplicative index (*V*)

$$V=\frac{X_{0}\times X_{AS}}{X_{A}\times X_{S}}$$

Where *X*_*0*_ is the odds ratio and/or relative risk for lung cancer among non-exposed to asbestos and non-smokers; *X*_*A*_ is the corresponding value for lung cancer among asbestos exposure in non-smokers; *X*_*S*_ is for lung cancer and smoking in those without asbestos-exposure; and *X*_*AS*_ is for lung cancer and co-exposure to asbestos and smoking. The synergy index (*S*) is an interaction on an additive scale. The interpretation is *S* = 1 suggests no interaction between asbestos exposure and smoking on lung cancer; *S* >1 suggests a positive interaction (synergism); and *S*<1 suggests a negative interaction (i.e., antagonism). For the multiplicative index (*V*), it can be interpreted as either: when *V* = 1, there is no interaction on the multiplicative scale; when *V* >1, the multiplicative interaction is positive; or when *V*<1, it is negative. Confidence intervals (CIs) were calculated using the method of Rothman, and Andersson et al. [38,45,46].

## Results

### Study Selection

**Fig 1:**
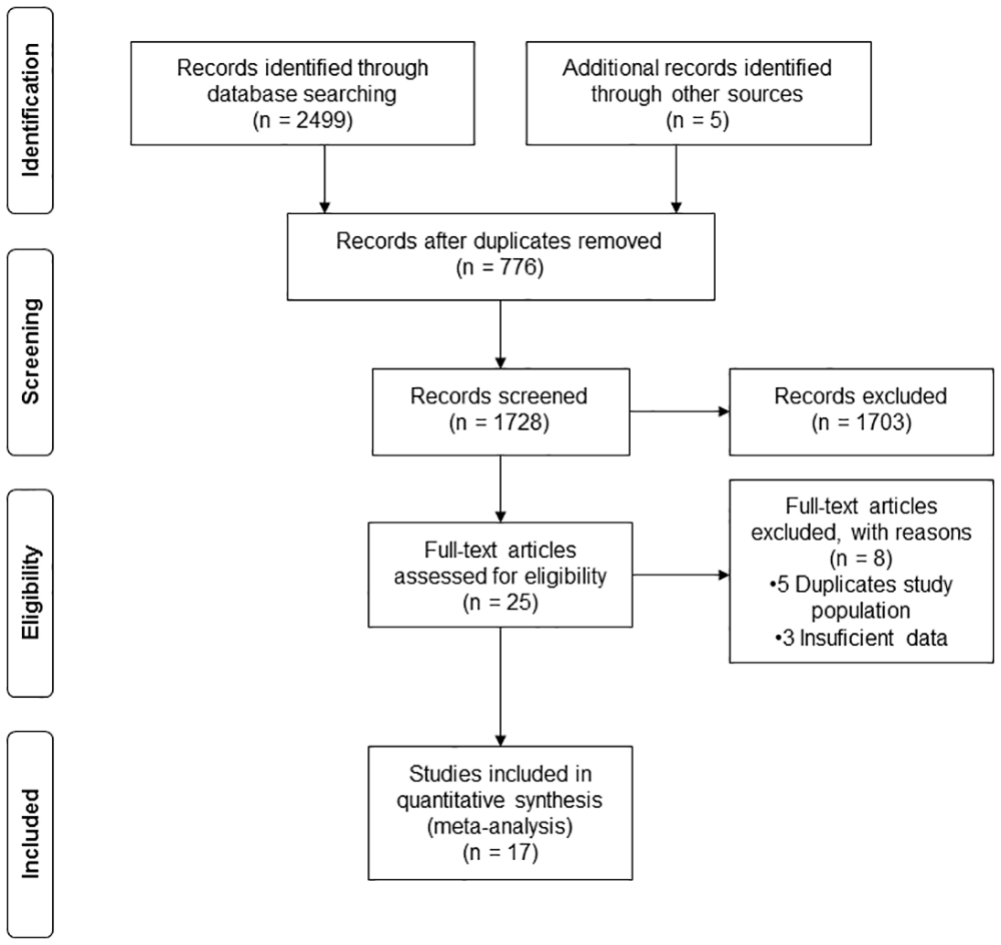
Summary of study search and selection.

We identified 2,499 records of which 2,479 were duplicated, irrelevant, review articles, case reports, non-human or experimental studies, or lacked lung cancer outcomes or lacking control groups, and were excluded. Five additional publications meeting the inclusion criteria were added from the bibliographies of the retrieved articles (Fig 1). In the final review of 25 studies, we excluded 5 studies [47–51] due to duplicate populations, and 3 studies [52–54] had insufficient data. Only one by Kjuus et al [55] was selected of three articles [47,48,55] which analyzed the same data. Case-control studies by Bovenzi (1992 and 1993) [49,56], the cohort studies of McDonald 1980 and Liddell 1984 [51,57]; and cohort studies of Klerk 1991 and Reid 2006 [26,50] also described the same populations of which the most recent [26,56,57] was selected. The Blot et al. study 1982 [52] did not report smoking status in asbestos-exposed populations. Finally, the studies of Hilt et al. 1986, and Markowitz et al. 1992 [53,54] were excluded because numbers of controls were missing. Therefore, a total of 17 studies (10 case-control and 7 cohort studies) were included for meta-analysis. The 13 included studies were identified using the search terms, and another 4 studies derived from their bibliographies.

### Study Characteristics

The characteristics and information of the included studies are shown in Table 1. The 10 case-control studies [22,25,27,55,56,58–62], contained 10,223 participants in all of which 4,768 were population-based controls, and 1,128 hospital-based controls. Seven cohort studies [23,24,26,57,63–65] had an aggregate of 64,924 participants, comprising of the 3,316 cases and 61,608 controls. In all the included studies asbestos exposure was occupational. Where reported, the average participant age was approximately 60 (range 40–80 y) for case control studies. Some [22,60] reported the type of asbestos used (tremolite or mixed asbestos), while the remaining eight [25,27,55,56,58,59,61,62] did not categorize the asbestos (Table 1). The settings for the exposure was occupational, either asbestos mines (one study [22]), ship building/repair (two studies [59,62]), textile production (one study [60]), and the remaining six [25,27,55,56,58,61] studies failed to specify. Environmental monitoring was measured by using the membrane filter method and were analyzed by phase contrast microscopy [25] but most studies relied on personal/telephone interview and/or questionnaire. Smoking habits of participants were quantified by personal/telephone interview and/or questionnaire. If the subject had already died, the appropriate information was sought from their next-of-kin or spouse (Table 2).

**Table 1:** Characteristics of studies included in the meta-analysis.

First author(year)	Location	Industrial type*	Asbestos type	Study design	Total population Case (n)	Total population Control (n)	NOS**
**Case-control studies (n = 10)**							
Martischnig(1977)	United Kingdom	Not specified	Not reported	Hospital-Based	201	201	6
Blot(1978)	Coastal Georgia, USA	Shipbuilding	Not reported	Hospital-Based	458	553	5
Blot(1980)	Coastal Virginia, USA	Shipyard	Not reported	Population-Based	319	341	6
Pastorino(1984)	Lombardy Northern, Italy	Manufacturing, textiles	Mixed	Population-Based	106	226	6
Kjuus(1986)	Southern Norway	Not specified	Not reported	Hospital-Based	176	176	7
Dave(1988)	Southeast Sweden	Not specified	Not reported	Hospital-Based	62	198	5
Bovenzi(1993)	Northeast Italy	Not specified	Not reported	Population-Based	516	561	6
Luce(2000)	New Caledonia, France	Mining & refining	Tremolite	Population-Based	103	110	6
Gustavsson(2002)	Stockholm, Sweden	Not specified	Not reported	Population-Based	768	1519	6
Villeneuve(2012)	8 locations, Canada	Not specified	Not reported	Population-Based	1618	2011	7
**Cohort studies (n = 7)**							
Berry(1972)	London, England	Asbestos factory	Not reported	Prospective	61	1678	6
Rubino(1979)	Balangero mine, Italy	Mining	Chrysotile	Prospective	12	54	7
Liddell(1984)	Quebec, Canada	Mining & milling	Chrysotile	Prospective	223	715	6
Berry(1985)	London, England	Asbestos factory	Not reported	Prospective	66	1268	6
Reid (2006)	Western Australia	Mining & milling	Crocidolite	Prospective	138	2595	7
Markowitz(2013)	USA	Insulator	Not reported	Prospective	2760	55161	8
Wang (2013)	China	Mining	Chrysotile	Prospective	56	137	7

*All studies are occupational exposures

**NOS = Newcastle Ottawa-Scale

**Table 2:** Descriptions of Asbestos Exposure and Smoking of Included Studies.

First author(year)	Measurement of exposure	Definition of asbestos exposure	Definition of asbestos exposure	Measurement of exposure	Definition of smoking	Definition of smoking
		Exposed	Non-exposed		Exposed	Non-exposed
**Case-control studies (n = 10)**						
Martischnig(1977)	Questionnaire	Occupational history (work in asbestos manufacturing or used asbestos)	No occupational history	Questionnaire	14 cigarettes/day or more	0–14 cigarettes/day
Blot(1978)	Personal interview	Occupational history (work in shipbuilding or used asbestos)	No occupational history (never work in shipbuilding)	Personal interview	10 cigarettes/day or more	<1/2 pack/day and stopped smoking at least 10 years
Blot(1980)	Personal interview	Occupational history (shipyard)	No occupational history (never work in shipyard)	Personal interview	10 cigarettes/day or more	<1/2 pack/day and stopped smoking at least 10 years
Pastorino(1984)	Personal interview	Exposed to asbestos only	Exposed other carcinogenic chemicals	Personal interview	10 cigarettes/day or more	0–9 cigarettes/day
Kjuus(1986)	Personal interview and questionnaire	Asbestos exposure at least 1 year or more and job title information	No exposure and no job title	Personal interview and questionnaire	10 cigarettes/day or more	0–9 cigarettes/day
Dave(1988)	Self-administered questionnaire and telephone interview	Occupational history (works related to asbestos)	Occupational history (other works)	Self-administered questionnaire and telephone interview	>80 cigarette-years	0 cigarette-years
Bovenzi(1993)	Personal interview	Occupational history (classified by job titles and asbestos exposure information)	No occupational history	Personal interview	>1 cigarette/day	No smoked
Luce(2000)	Personal interview	Occupational history (classified by expert assessment)	No occupational history	Personal interview	>20 pack-years	< 20 pack-years
Gustavsson(2002)	Questionnaire, telephone interview and environmental measurement	Occupational history and asbestos exposure > 0 fiber-years	No occupational history and asbestos exposure 0 fiber-years	Questionnaire and telephone interview	>1 cigarette/day	No smoked
Villeneuve(2012)	Questionnaire	Occupational history (classified by concentration, frequency and reliability)	No occupational history	Questionnaire	10 pack-years or more	< 10 pack-years
**Cohort studies (n = 7)**						
Berry(1972)	Questionnaire	Occupational history	No occupational history	Questionnaire	smoked	No smoked
Rubino(1979)	Environmental measurement	Occupational history (mining)	No occupational history	Personal interview	smoked	No smoked
Liddell(1984)	Environmental measurement	Cumulative exposure >100 fiber/year	Cumulative exposure 0–100 fiber/year	Questionnaire	>1 pack-years	0 pack-years
Berry(1985)	Questionnaire	Occupational history	No occupational history	Questionnaire/interview	smoked	No smoked
Reid(2006)	Questionnaire and environmental measurement	Occupational history	No occupational history	Questionnaire	Smoked and ex-smoked < 20 years	No smoked and ex-smokers > 20 years
Markowitz(2013)	Clinical method (x-ray and spirometry)	Occupational history (insulation)	No occupational history	Not reported	smoked	No smoked
Wang(2013)	Environmental measurement	Cumulative exposure >20 fiber-year/ml	Cumulative exposure <20 fiber-year/ml	Questionnaire/interview	smoked	No smoked

There were seven cohort studies, and all of these collected asbestos exposure data prospectively and also prospectively for smoking data in six studies and retrospectively in one [64]. The mean follow-up period of cohort studies was 19.3 yr. Exposure was to chrysotile in three studies [23,57,65], one study to crocidolite [26], and the asbestos type was unspecified in remaining three studies [24,63,64] (Table 1). Four studies [23,26,57,65] were from mining and three studies [24,63,64] originated from factories making asbestos products. Workplace asbestos exposure was assessed by lung histology, counting fibers trapped by midget impingers or membrane filters [23,57,65], a long-duration personal konimeter [26], or postal questionnaires [63,64]. Only one study assessed exposure by chest X-ray radiographs and a low FEV1 by spirometry [24]. Smoking was assessed by interviewing or questionnairing the workers or their next-of-kin (Table 2). Diagnosis of lung cancer was confirmed by histological examination of lung biopsies, chest X-ray, CT scan, MRI, bronchoscopy, or thoracoscopy. Most studies classified lung cancer using the International Classification of Diseases (ICD), published by the World Health Organization (Table 3).

**Table 3:** Descriptions of Outcome of Included Studies.

Author (Year)	Case confirmation method	Diagnosis period	Lung cancer classification	Control matching	Period of exposure or employment
**Case-control studies (n = 10)**					
Martischnig(1977)	Radiography, bronchoscopy or thoracotomy	1972–1973	Not reported	Age (±2 years)	1–5 years and 6 years and over
Blot(1978)	By physician	1970–1976	ICD 8 162.1	Sex, race, age (±2 years)	6 months or more
Blot(1980)	By physician	1976	ICD 162.1	Race, age, death year, city of residence	6 months or more
Pastorino(1984)	By physician	1976–1979	Not reported	Age (±2 years)	6 months or more
Kjuus(1986)	By examination of histology		ICD 162–163	Age (±5 years)	1979–1983
Dave(1988)	Not reported	1980–1982	ICD 162–163	Age, sex	Not reported
Bovenzi(1993)	Histology, autopsy reports		ICD 9^th^ 162	Age (±2 years)	Not reported
Luce(2000)	Clinical, radiological & endoscopic	1993–1995	ICD for oncology topography code 160–162,148	Sex, age (±5 year)	Not reported
Gustavsson(2002)	Not reported	1985–1990	ICD 7^th^ 162.1	Age (±5 year) and year of inclusion study (1985–1990)	1969–1973
Villeneuve/2012	By examination of histology	1994–1997	ICD 9^th^ 162	Age, sex	At least 12 months
**Cohort studies (n = 7)**					
Berry(1972)	By examination of histology	Not reported	ICD 162,163	Not reported	Men 1933–1955 Women 1936–1942
Rubino(1979)	By physician	1957	ICD 7 162/163	Age (±1 year)	1930–1965
Liddell(1984)	Not reported	Not reported	ICD 7^th^	Not reported	1966–1975
Berry(1985)	By examination of histology	Not reported	The Office of Population Censuses and Surveys	Not reported	Men 1933–1955 Women 1936–1942
Reid(2006)	By physician	2000 and 2002	ICD-0 2^nd^ edition categories c33.9-c34.9	Sex, age (±5 years)	1979–2002
Markowitz(2013)	By chest radiographs	1981 and 1983	ICD-9 code 162 (1981–1998) and ICD-10 codes C-33 and C-34 (1999–2008)	Not reported	1982–2008
Wang(2013)	By pathology or biopsy	The first two decades	The Chinese Radiographic Diagnosis Criteria of Pneumoconiosis	Not reported	1981–2006

ICD stands for International Classification of Diseases

### Quality Assessment

The methodological quality of case-control studies was summarized as a mean NOS of 6 (range 5–7) and a score of 6.7 (range 6–8) for cohort studies (Table 1).

### Quantitative Synthesis

**Fig 2:**
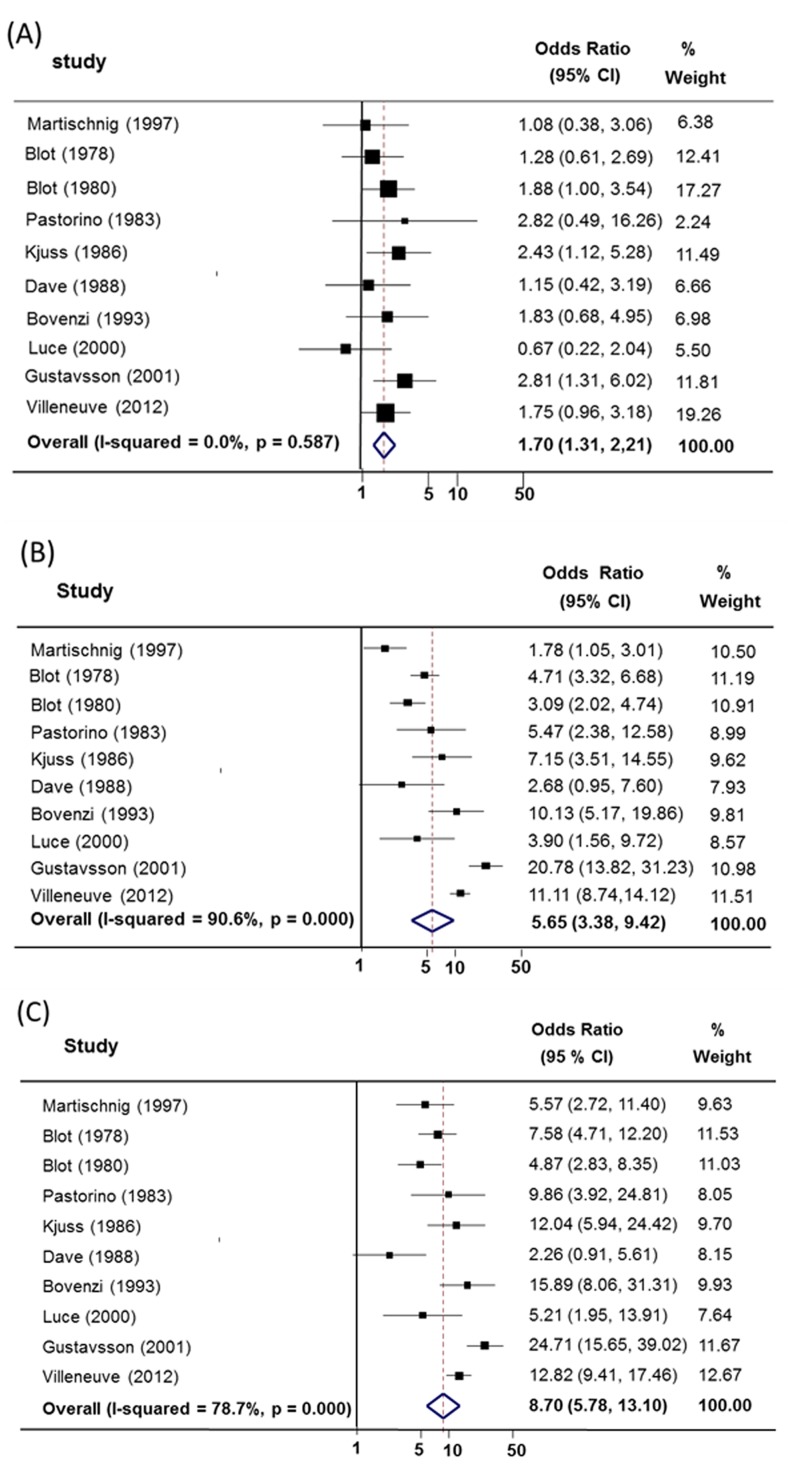
Random-effects meta-analysis of the synergistic effect between asbestos exposure and smoking cause lung cancer- Case control studies. (A) Summary odds ratio of asbestos-exposed and non-smoking (A+S-) compared with not asbestos-exposed and non-smoking (A-S-). (B) Summary odds ratio of non-exposure to asbestos and smoking (A-S+) compared with not asbestos-exposed and non-smoking (A-S-). (C) Summary odds ratio of asbestos-exposed and smoking (A+S+) compared with not asbestos-exposed and non-smoking (A-S-).

**Fig 3:**
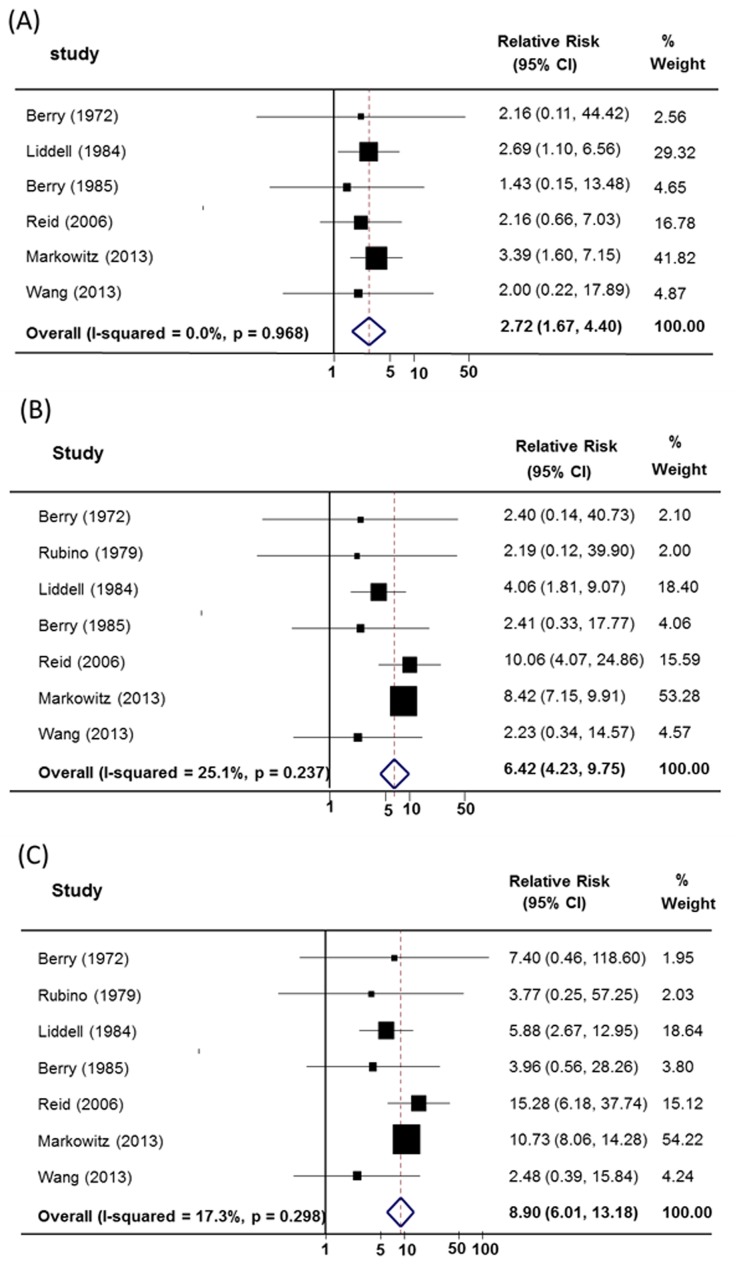
Random-effects meta-analysis of the synergistic effect between asbestos exposure and smoking cause lung cancer- Cohort study. (A) Summary relative risk of asbestos-exposed and non-smoking (A+S-) compared with not asbestos-exposed and non-smoking (A-S-). (B) Summary relative risk of non-exposure to asbestos and smoking (A-S+) compared with not asbestos-exposed and non-smoking (A-S-). (C) Summary relative risk of asbestos-exposed and smoking (A+S+) compared with not asbestos-exposed and non-smoking (A-S-).

(i) **Case-control studies:** A random-effects meta-analysis of 10 studies [22,25,27,55,56,58–62] revealed associations between asbestos exposure and/or smoking, and developing lung cancer. The summary odds ratio of (A+S-) workers compared with (A-S-) workers was 1.70 (95% CI = 1.31–2.21). The summary odds ratio of (A-S+) workers compared with (A-S-) was 5.65 (95% CI = 3.38–9.42). Additionally, the summary odds ratio of (A+S+) workers compared with (A-S-) workers was 8.70 (95% CI = 5.78–13.10). Evidence of heterogeneity was found in A-S+/A-S- and A+S+/A-S- groups (*I* ^*2*^ = 90.6%, *p* = 0.000 and *I* ^*2*^ = 78.7%, *p* = 0.000) (Fig 2A–2C). As shown in Table 4, the results of subgroup analyses according to different characteristics are in close agreement with our major findings. Such heterogeneity probably arises from the differing interaction effects across varying levels of smoking exposure. We stratified studies with similar smoking classification by subdivision into 3 levels: non-smokers (non-smoking or light smoking), moderate smokers (1–19 cigarettes/day) and heavy smokers (>20 cigarettes/day) (Table 5). There were no differences between non-smokers 2.63 (95% 1.43–4.83) and light smokers 2.63 (95% 1.57–4.42) for exposed-asbestos group. But for both subgroups, the moderate and heavy smoking categories showed elevated odds ratios with asbestos exposure.
*Publication bias*: Begg’s funnel plot and Egger’s test were performed to assess publication bias of the literature. Publication bias for (i) A+S- was *p* = 0.437 (Begg’s test), and 0.659 (Egger’s), (ii) A-S+ was *p* = 0.252 (Begg’s test), and 0.362 (Egger’s), and (iii) A+S+, *p* = 0.154 (Begg’s test) and 0.294 (Egger’s test) suggesting no bias. Funnel plots suggested evidence of publication bias. There was asymmetry of funnel plots accordant with high heterogeneity studies (A-S+ and A+S+). However, trim and fill analysis showed that the overall odds ratios were unchanged (data shown in supplement, S1 Fig).
(ii) **Cohort studies:** Seven studies [23,24,26,57,63–65] were included in our primary analysis (Fig 3A–3C). The summary relative risks for lung cancer in the cohort studies of (A+S-) workers was 2.72 (95% CI = 1.67–4.40), (A-S+) workers was 6.42 (95% CI = 4.23–9.75), and for (A+S+) workers was 8.90 (95% CI = 6.01–13.18) compared with (A-S-) workers. The results of the cohort studies are consistent with the analysis of the case-control studies. Evidence of heterogeneity was not found in cohort studies (*I* ^*2*^ = 0.0%, *p* = 0.968, *I* ^*2*^ = 25.1%, *p* = 0.237 and *I* ^*2*^ = 17.3%, *p* = 0.298). In addition, case-control studies estimates of the combined effect of asbestos and smoking on lung cancer risk were in concordance with those from cohort studies.
*Publication bias*: Evaluation of publication bias for A+S-, A-S+ and A+S+ are Begg’s test (*p* = 0.063) Egger’s test (*p* = 0.079), Begg’s test (*p* = 0.026) Egger’s test (*p* = 0.065) and Begg’s test (*p* = 0.118) Egger’s test (*p* = 0.254), respectively. These results did not indicate a potential for publication bias when using funnel plots (data shown in supplement, S2 Fig).

**Table 4:** Effect of the Exposure to Asbestos (A) and/or Cigarette Smoking (S) on Lung Cancer Risk.

Groups	No. of studies		ORs and RRs* (95% CI)	ORs and RRs* (95% CI)	ORs and RRs* (95% CI)	*P* for heterogeneity	*P* for heterogeneity	*P* for heterogeneity	*I* ^*2*^ (%)	*I* ^*2*^ (%)	*I* ^*2*^ (%)
		Reference**	A	S	A and S	A	S	A and S	A	S	A and S
**Case-control studies**											
***Geographic area***											
USA	2	1.00	1.60 (0.99–2.59)	3.89 (2.58–5.86)	6.19 (4.01–9.54)	0.435	0.136	0.228	0.0	55.0	31.3
Europe	7	1.00	1.71 (1.15–2.54)	5.63 (2.49–12.71)	8.89 (4.77–16.56)	0.339	0.000	0.000	11.9	90.4	80.3
***Study design***											
Population Based	6	1.00	1.83 (1.32–2.55)	7.60 (4.09–14.11)	10.92 (6.54–18.22)	0.464	0.000	0.000	0.0	89.7	79.2
Hospital Based	4	1.00	1.49 (0.97–2.29)	3.60 (1.94–6.69)	6.19 (3.47–11.06)	0.501	0.005	0.034	0.0	76.8	65.3
**Cohort studies**											
***Asbestos type***											
Chrysotile	3	1.00	2.58 (1.13–5.89)	3.58 (1.75–7.33)	5.04 (2.50–10.18)	0.807	0.798	0.685	0.0	0.0	0.0
Not reported	3	1.00	3.05 (1.53–6.08)	7.33(4.18–12.85)	10.47 (7.90–13.88)	0.736	0.326	0.501	0.0	10.8	0.0

*Odds ratios is for case-control, relative risk is for cohort study

** Reference is equal one as control group

**Table 5:** Effect of the Exposure to Asbestos (A) and/or Cigarette Smoking (S) on Lung Cancer Risk in Case-Control Studies, Stratified by smoking levels.

Smoking level	No. of studies	ORs (95% CI)	ORs (95% CI)	ORs (95% CI)	*P* for heterogeneity	*P* for heterogeneity	*P* for heterogeneity	*I* ^*2*^ (%)	*I* ^*2*^ (%)	*I* ^*2*^ (%)
		A	S	A and S	A	S	A and S	A	S	A and S
Non smokers	2^[^ ^25^ ^,^ ^55^ ^,^ ^56^ ^]^	2.63 (1.43–4.83)	-	-	0.785	-	-	0.0	-	-
1–19 cigarettes/day	2	-	9.98 (3.44–28.96)	15.38 (7.34–32.24)	-	0.010	0.083	-	85.1	66.8
>20 cigarettes/day	2	-	25.41 (8.96–72.00)	30.31 (15.77–58.25)	-	0.011	0.168	-	84.4	47.5
0–9 cigarettes/day	3^[^ ^25^ ^,^ ^55^ ^,^ ^60^ ^]^	2.63 (1.57–4.42)	-	-	0.964	-	-	0.0	-	-
10–19 cigarettes/day	3	-	8.54 (2.76–14.76)	13.13 (7.34–32.24)	-	0.000	0.019	-	87.6	74.9
>20 cigarettes/day	3	-	15.76 (4.36–56.94)	25.94 (11.94–56.39)	-	0.000	0.119	-	87.7	53.0

### Interaction between asbestos exposure and cigarette smoking

Evaluation of interaction is summarized in Table 6. All 17 studies provided data which enabled evaluation of the joint effects of co-exposure of both asbestos and cigarette smoking on the risk of lung cancer. For case-control studies, the interaction index of synergy (*S*) and multiplicative index (*V*) were 1.44 (95% CI = 1.26–1.77) and 0.91 (95% CI = 0.63–1.30), respectively, with corresponding values for the cohort studies of 1.11 (95% CI = 1.00–1.28) and 0.51 (95% = 0.31–0.85). These results suggest that the interaction between asbestos exposure and smoking can be a positive interaction on the additive scale (an additive synergistic effect). There was a suggestion of a negative multiplicative interaction for both case-control and cohort studies. Notably our results do not show a multiplicative effect between the two known human carcinogens.

**Table 6:** Synergy and Multiplicative Indices between Asbestos Exposure and Cigarette Smoking.

Overall risk estimates	Reference	Asbestos	Smoking	Asbestos and smoking	Interaction index*	Interaction index*
					synergy	multiplicative
Odds Ratio	1.00	1.70(1.31–2.21)	5.65(3.38–9.42)	8.70(5.78–13.10)	1.44 (1.26–1.77)	0.91(0.63–1.30)
Relative Risk	1.00	2.72(1.67–4.40)	6.42(4.23–9.75	8.90(6.01–13.18)	1.11 (1.00–1.28)	0.51(0.31–0.85)

* Rothman synergy index

## Discussion

Our results demonstrate a positive synergistic interaction on an additive scale between asbestos exposure and cigarette smoking in workers developing lung cancer (Table 6). Employees exposed to asbestos and having a history of smoking have a higher risk of developing lung cancer than those only exposed to one risk (either smoking or asbestos alone). In contrast, the multiplicative index for case-control studies was close to 1.0, although for cohort studies, a negative multiplication interaction is suggested (*V* = 0.51, 95%CI = 0.31–0.85).

Some data suggests that smoking does not enhance mesothelioma [66], which implies that the synergistic lung cancer risk arises from the two carcinogens interacting in the same lung tissue. There are several mediators contributing to cigarette smoke and asbestos-induced lung diseases. Both smoking [67] and asbestos [68] elicit chronic inflammation, which is central to tumorigenesis and is augmented through reduced active immunity, increased infections, and compromised tumor surveillance [69,70]. Tobacco smoke causes inflammation through a vast array of chemical and particulate irritants. Mineral fibers are inflammatory primarily through activation of Nod-like receptor-family protein 3 (NLRP3) of inflammasomes in tissue macrophages. Asbestos fibers evoke vain attacks by macrophages ensuring their continual activation while also adversely affecting function of other immune cells [71,72]. Symptoms of inflammation include oxidative stress, which is worse in blue asbestos (amosite, crocidolite, tremolite) containing Fe ions which generate additional reactive species through Fenton catalysis [73]. The prolonged bio-persistence of these amphiboles further contributes to their greater carcinogenicity than chrysotile and other mineral fibers. Tobacco smoke also contains multiple carcinogens (e.g., 4-(methylnitrosamino)-1-(3-pyridyl)-1-butanone or NNK, 1,3-butadiene, ethylene oxide, chromium, polonium-210, arsenic, ethyl carbamate, and hydrazine) that directly interact with DNA [74]. Thus, the common localized inflammatory actions of tobacco smoke and asbestos readily explains additive effects, while the additional actions (direct carcinogenesis and Fenton catalysis) of each insult could account for the additive synergistic interaction.

The present study has some limitations which are mostly inherent in this type of study.

Odds ratios were roughly estimated from the included studies where the measurement methods used and exposure classification varied between studies. For example there were several studies claiming that the duration of asbestos exposure was the same as the period of employment in the workplace. Therefore, short duration jobs reduce the validity and reliability of questionnaires about occupational history. Some studies [58,60,61] did not provide estimates of adjusted risks (age, sex, etc.). The methods used to quantitate exposures to asbestos and cigarette smoke were arbitrary and varied across studies. The type of asbestos used was usually not stated. The diagnosis for lung cancer used different criteria (by physician, chest x-ray, radiography, or information taken from the death certificate). In contrast, other studies have objective exposure and clinical criteria (e.g., Markowitz et al. [24]). The type of lung cancer was rarely stated or even whether mesothelioma was excluded but mesothelioma was never explicitly included. Some case-control studies [55,59] used control populations who had other diseases (e.g., myocardial infarction, bladder cancer, other malignant neoplasms or other lung disease). Most of these diseases are also smoking-related. Nevertheless, all case-control studies endeavored to match controls for confounders. Some studies have data derived from recalling events that took place 10 years or more before the interview/questionnaire, which raises the issue of recall bias and misclassification. Subgroup analysis by smoking level retained high heterogeneity (Table 5) probably due to different methods of data collection and measurement, uncertain duration of smoking (only daily number of cigarettes smoked quoted).

Nevertheless, our study has some strength. It includes new data and the selection criteria complied with the PRISMA and MOOSE guidelines to perform the first systematic review and meta-analysis. Our analysis differed from previous analyses because (i), the strict selection criteria and heterogeneity testing, (ii) testing for statistical interaction (additive and multiplicative). Most studies randomly enrolled greater numbers of control subjects from hospital registers or health authority databases thus reducing selection bias. One study [59] excluded participants who provided incomplete questionnaire data, were non-responders, or who had emigrated from the area. These unavoidable variations in the study population and diverse methods utilized readily explain the substantial heterogeneity we detected.

While the most dangerous asbestos types are no longer used, other siliceous fibers and chrysotile (in developing nations) are still incorporated into many building products without clear long-term health assessments in humans. Workers exposed to chrysotile showed increased risk of lung cancer (Table 4) [75]. The scientific rigor of cohort studies has improved since the early asbestos work. However, the long latencies for asbestos-induced neoplasms [76] make retrospective study the only practical protocol. Cigarette smoke inhalation and hence airway exposure can be accurately assessed (cigarette numbers, inhalation, filters). However, our study reiterates the difficulty in accurately assessing actual airway exposure to asbestos and was best assessed in the Markowitz et al. study [24]. Personal monitors provided the best indication of exposure but ultimately, only random sputum fiber counts by public health agencies can provide unbiased and accurate measures of exposure. Another problem highlighted by Markowitz et al. [24] and our study is accurately diagnosing the end-stage pathology. Again, monitoring by independent public health authorities is the mechanism most likely to yield accurate reporting. In addition, potential confounders including life-style and especially local air quality data need collecting for the same cohorts.

## Conclusion

The present meta-analysis collected and synthesized data currently available and revealed a positive interaction on an additive scale between asbestos exposure and smoking, while showing little evidence of an interaction on a multiplicative scale. The combined effect of asbestos exposure with moderate and heavy smoking in lung cancer suggested a strong positive interaction on an additive scale, i.e., an additive synergism.

## Supporting Information

S1 Fig Funnel plot for 10 case-control studies of relationship between asbestos and cigarette smoking on lung cancer with subjects whom are exposed to asbestos and non-smokers (A), subjects whom are not exposed to asbestos and smokers (B) and subjects whom are exposed to asbestos and smokers (C). (DOCX)

S2 Fig Funnel plot for 7 cohort studies of relationship between asbestos and cigarette smoking on lung cancer with subjects whom are exposed to asbestos and non-smokers (A), subjects whom are not exposed to asbestos and smokers (Be) and subjects whom are exposed to asbestos and smokers (C). (DOCX)

S1 PRISMA Checklist PRISMA 2009 Checklist. (DOC)
