# Supplementary material for: Additive Synergism between Asbestos and Smoking in Lung Cancer Risk: A Systematic Review and Meta-Analysis
Source: PLoS One. 2015 Aug 14;10(8):e0135798. doi: 10.1371/journal.pone.0135798 (PMC4537132; doi:10.1371/journal.pone.0135798)
Supplement: S2 Fig — (DOCX) [file pone.0135798.s002.docx]

**
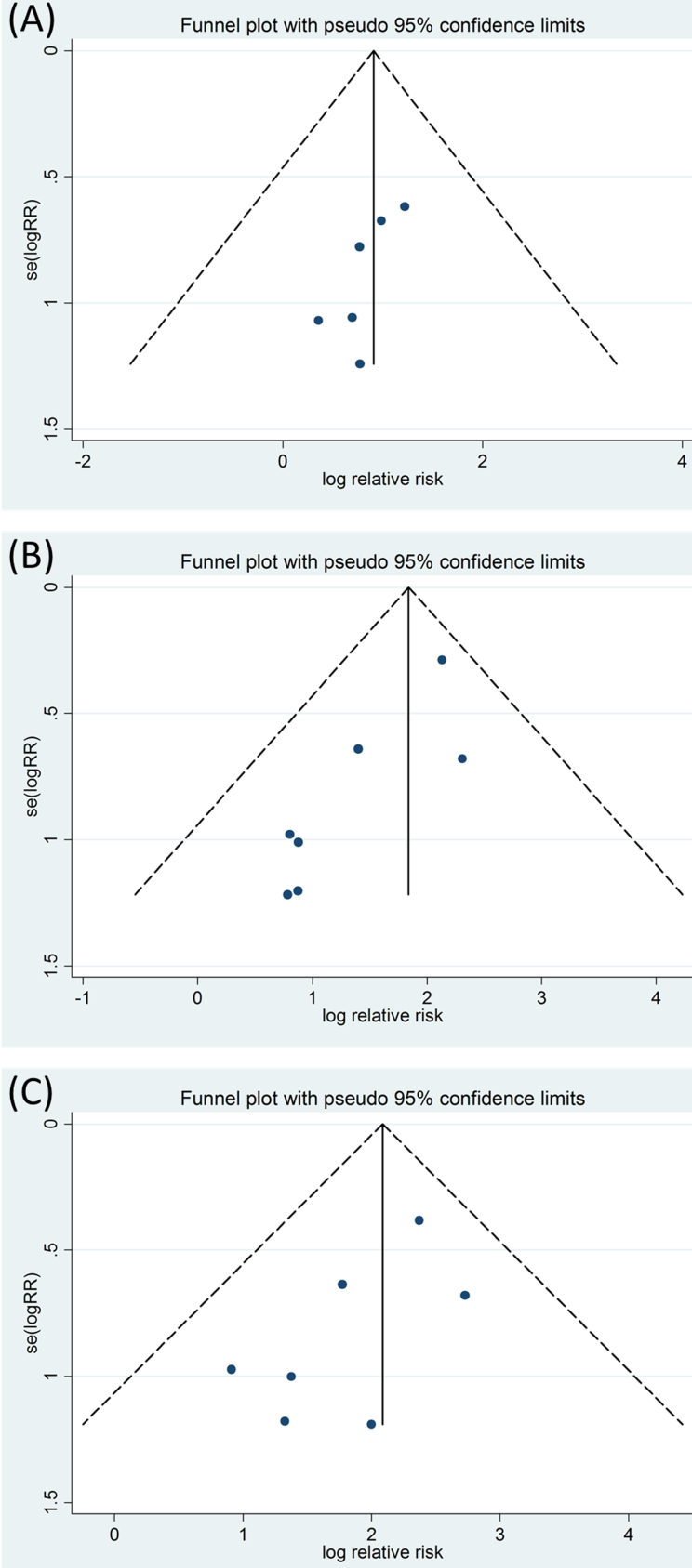
**

**S2 Figure:** Funnel plot for 7 cohort studies of relationship between asbestos and cigarette smoking on lung cancer with subjects whom are exposed to asbestos and non-smokers (A), subjects whom are not exposed to asbestos and smokers (B) and subjects whom are exposed to asbestos and smokers (C).
